# Hesitancy to Receive the Second COVID-19 Vaccine Booster Dose among Older Adults in Hong Kong: A Random Telephone Survey

**DOI:** 10.3390/vaccines11020392

**Published:** 2023-02-08

**Authors:** Paul Shing-fong Chan, Marco Lok-tin Lee, Yuan Fang, Fuk-yuen Yu, Danhua Ye, Siyu Chen, Joseph Kawuki, Xue Liang, Zixin Wang

**Affiliations:** 1 Jockey Club School of Public Health and Primary Care, The Chinese University of Hong Kong, Hong Kong, China; 2 Department of Health and Physical Education, The Education University of Hong Kong, Hong Kong, China

**Keywords:** vaccine hesitancy, second COVID-19 booster dose, older adults, factors, China

## Abstract

A second COVID-19 vaccine booster dose is effective and safe for older adults. This study investigated hesitancy to take up a second COVID-19 vaccine booster dose and its determinants among older adults in Hong Kong. Participants were Chinese-speaking community-dwelling adults aged 65 years or above. Telephone numbers were randomly selected from up-to-date telephone directories. A total of 370 participants completed the telephone survey. Logistic regression models were fitted for data analysis. Among the participants, half (52.4%) were hesitant to receive the second COVID-19 vaccine booster dose. After adjustment for significant background characteristics, perceived benefits (AOR: 0.50, 95%CI: 0.42, 0.60), cues to action (AOR: 0.39, 95%CI: 0.30, 0.52), and perceived self-efficacy (AOR: 0.37, 95%CI: 0.21, 0.66) of receiving the second booster dose were associated with lower vaccine hesitancy. Perceived barriers (AOR: 1.23, 95%CI: 1.12, 1.34) and vaccine fatigue (tired of receiving repeated COVID-19 vaccination) (AOR: 1.90, 95%CI: 1.52, 2.38) were associated with higher vaccine hesitancy. Level of hesitancy to receive the second booster dose was high among older adults in Hong Kong. Health authorities should address vaccine fatigue and modify perceptions related to the second booster dose.

## 1. Introduction

Globally, the ongoing pandemic of coronavirus disease 2019 (COVID-19) is still a severe public health issue. As of 12 December 2022, there have been 646 million confirmed cases and 6.6 million deaths caused by COVID-19 worldwide [1]. Increasing age is a leading risk factor of severe COVID-19 cases and mortality [2]. In Hong Kong, where this study was conducted, the cumulative number of COVID-19 associated deaths was 11,131 on 16 December 2022, and over 90% of them were individuals aged 60 years or above [3]. The majority (72.5%) of the COVID-19 associated deaths among older adults in Hong Kong occurred among those who had never received any COVID-19 vaccines [3].

COVID-19 vaccination is one of the most cost-effective measures to control the pandemic. It is proven to be effective and safe in preventing deaths and other severe consequences caused by COVID-19 among older adults [4,5,6]. However, there are concerns about waning protection against COVID-19 after completing the two-dose primary vaccination series [7,8]. The waning of protection is even faster in older adults [9,10]. Administering an additional booster dose of vaccine is proven to restore immune response against COVID-19 and successfully decrease the rate of infection and hospitalization [8,11]. However, waning immunity to the first booster dose and evolving highly contagious new variants of SARS-CoV-2 have led to the consensus recommendation of prioritizing high-risk groups, such as older adults, for a second booster dose [8,11,12].

A second booster dose could help increase protection levels, especially for individuals in high-risk groups, such as older adults. Numerous studies demonstrated that a second booster dose was effective in reducing the risk of COVID-19-related outcomes (including those caused by the new variants, Delta and Omicron), such as polymerase chain reaction-confirmed infection, symptomatic infection, severe illness, hospitalization, emergency department and urgent care counters and deaths [13,14,15,16,17,18]. A prospective observational trial showed that 65% of subjects reported no side effects after receiving the second booster dose [19]. The most frequently reported adverse reactions included fatigue, headache, and muscle pain, and these mild adverse reactions resolved within three days [19]. Moreover, according to a safety monitoring report from the United States, the administration of a second booster dose did not result in any unexpected safety signals, and 95% of the adverse events reported to the surveillance system were non-serious [20].

Based on the vaccine effectiveness and safety of receiving the second COVID-19 vaccine booster dose, on 18 August 2022, the World Health Organization (WHO) issued a practice statement and recommended countries consider a second COVID-19 vaccine booster dose in the populations at risk, including older people [21]. Health authorities from different countries also endorsed the second booster dose for older adults, with the age eligibility threshold slightly different across countries, commonly starting from 50 or 60 years old [22,23,24,25,26]. In April 2022, the Hong Kong government started providing the second COVID-19 vaccine booster dose to people aged 60 years or above [27]. The age threshold was further lowered to 50 years old in August 2022, and then expanded to all adults [28]. People can choose either the BNT162b2 mRNA vaccine, or the Sinovac-CoronaVac inactivated vaccine as a second booster dose.

Despite the promising evidence of COVID-19 vaccine effectiveness and safety, vaccine hesitancy is still a significant threat to the rollout of COVID-19 vaccine booster doses. Previous studies showed that older adults were less willing to receive the primary COVID-19 vaccination series and first booster dose than the younger groups in China [29,30,31,32]. The studies identified some factors associated with hesitancy to complete the primary vaccination series or first booster dose among older adults. The barriers included the belief that they were ineligible for vaccination due to certain illnesses, concerns about vaccine safety and adverse side effects, concerns about efficacy, limitation on movements, and low level of perceived benefit [29,30,31,32]. In contrast, being male, having higher education level, and high level of perceived susceptibility were found to be facilitators [29,30,31,32]. Two studies from Greece investigated vaccine hesitancy on the second booster dose in nurses and the general population [33,34]. However, no study has investigated the hesitancy to receive the second booster dose and its associated factors among older adults.

Vaccine fatigue refers to people’s inaction toward vaccine instructions due to perceived burden and burnout [35]. The need for COVID-19 vaccine booster doses and the rapidly changing recommendations and guidelines may increase vaccine fatigue, thus increasing vaccine hesitancy. In Pakistan, 83.3% of COVID-19 vaccination recipients had vaccine fatigue after the primary vaccination series [36]. The present study also investigated the association between vaccine fatigue and hesitancy to receive the second COVID-19 vaccine booster dose among older adults. 

COVID-19 vaccinations and booster doses are hot topics on different media. Exposure to information related to COVID-19 vaccination influenced people’s decision to take up such vaccines. The general population in China with a higher frequency of exposure to positive information supporting COVID-19 vaccination were more likely to complete the primary vaccination series [37]. It is possible that such exposure would have a similar effect on the willingness to receive COVID-19 vaccine booster doses among older adults. Thoughtful consideration of the veracity of the exposed information was also significantly associated with higher COVID-19 vaccination uptake among the general population and ethnic minorities in China [37,38]. Such practice may mitigate the negative impact of misinformation related to COVID-19 and COVID-19 vaccination. Therefore, the present study also examined the association between vaccine hesitancy and exposure to information about COVID-19 vaccine booster doses through social media. 

It is essential to understand vaccine hesitancy to receive the second COVID-19 booster dose and its associated factors among older adults in order to design effective health promotion strategies. However, there is a dearth of studies investigating the hesitancy to receive the second booster dose among older adults. To address the knowledge gaps, this study investigated the vaccine hesitancy to receive the second COVID-19 vaccine booster dose among older adults in Hong Kong, China. In addition, we examined the factors associated with the vaccine hesitancy to receive the second booster dose.

## 2. Materials and Methods

### 2.1. Study Design

**Figure 1:**
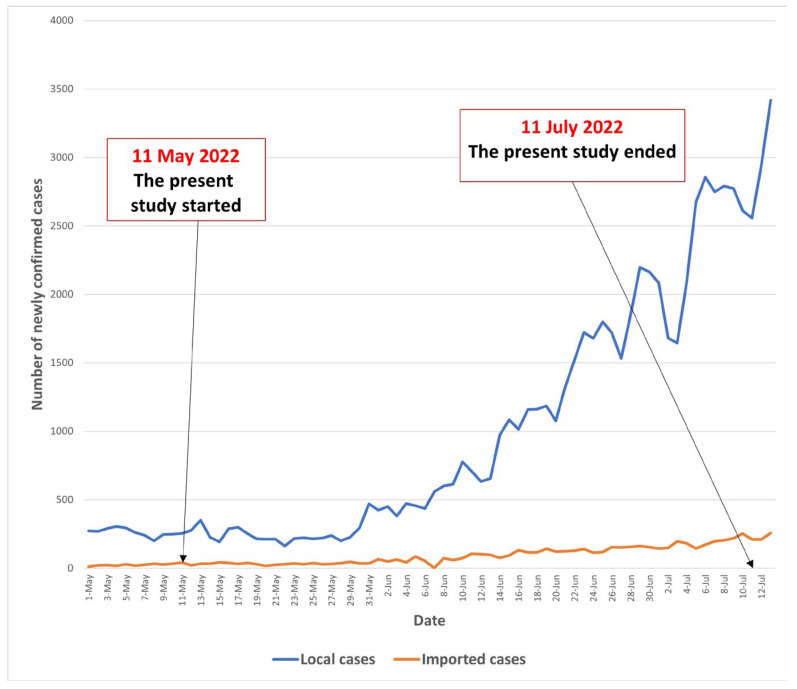
The COVID-19 situation in Hong Kong during the study period.

We conducted a random telephone survey among community-dwelling Chinese-speaking adults aged 65 years or above in Hong Kong between 11 May 2022 and 11 July 2022. The number of local daily-confirmed COVID-19 cases was 254 on 11 May 2022 and slowly increased to 2558 on 11 July 2022. The COVID-19 situation in Hong Kong during the study period is illustrated in Figure 1.

### 2.2. Participants and Data Collection

The inclusion criteria of the participants were: (1) community-dwelling Chinese-speaking individuals aged 65 years or above, and (2) having a Hong Kong ID card. Those who were not able to communicate effectively with the study interviewers were excluded.

Simple random sampling was used. All the household telephone numbers (about 350,000) listed on the up-to-date telephone directories in Hong Kong were inputted into an Excel sheet. Using the random selection function in the software, about 4000 telephone numbers were randomly selected. Experienced interviewers conducted the telephone interview during 6:00–10:00 p.m. on weekdays and 2:00–9:00 p.m. on Saturdays to prevent under-sampling of individuals who worked on weekdays. Each number was called up to 5 times at different timeslots. Households were regarded as “non-valid” (i.e., absence of an eligible participant) if no one answered the call after five times of calling. To avoid clustering effects, if there was more than one individual in a household aged 65 or more, the one with a birthday closest to the survey date was invited to join the study. Interviewers screened the eligibility of prospective participants and provided a study briefing to participants. Verbal informed consent was obtained from all participants. Prior to the interview, the interviewers used a checklist to confirm that the participant was fully informed about the study. There were six parts on the checklist: (1) questions to confirm eligibility, (2) scrips about study information, (3) the interviewers confirming they had introduced the research purpose, research process, main content of the survey, time required for completing the survey, rights of the participant, that non-participation would not affect the use of any services, and confidentiality of the research data, (4) the interviewers confirming that the participant fully understood the above contents, (5) the interviewers confirming that the participant verbally expressed his/her willingness to participate in the study, and (6) signature of the interviewers. No incentive was offered for study participation. The whole survey took around 20 min to complete. The same data collection method was used in a number of published studies [39,40,41,42]. We called 3840 households, 625 households had an eligible participant, 255 refused to participate in the study, and 370 completed the telephone survey. The response rate was 59%. Ethics approval was obtained from the Survey and Behavioral Research Ethics Committee of the Chinese University of Hong Kong (SBRE-19-187).

### 2.3. Measures

#### 2.3.1. Design of the Questionnaire

A panel of researchers in public health, behavioral health, and vaccination behaviors was formed to design the questionnaire used in this study. The questionnaire was pilot tested among 10 older adults to assess clarity and readability. All the older adults participating in the pilot study indicated that the items of the questionnaire were easy to understand and the length of the questionnaire was acceptable. These older adults did not participate in the actual survey. The panel finalized the questionnaire based on their comments.

#### 2.3.2. Background Characteristics

Information on socioeconomic characteristics was collected, such as gender, age, educational level, relationship status, employment status, household income level, whether living alone, and whether they were receiving Comprehensive Social Security Assistance (CSSA). Participants also reported their current health condition and history of seasonal influenza vaccination, pneumococcal vaccination, and COVID-19 infection.

#### 2.3.3. COVID-19 Vaccination Uptake and Hesitancy to Receive the Second COVID-19 Vaccine Booster Dose

Information on the vaccination status of the participants, including the number of vaccine doses, the timing and type(s) of vaccine received and any side effects after vaccination, was collected. Vaccine hesitancy was measured using the same definition as in published studies [43,44]. It was assessed by first asking the participants, “Have you received the second COVID-19 vaccine booster dose (the fourth dose)?”. If they answered “no”, they were further asked the following question, “How likely is it that you will receive the second COVID-19 vaccine booster dose (the fourth dose) in the future 6 months?”. Answer options were on a 5-point Likert scale: 1 = very unlikely, 2 = unlikely, 3 = neutral, 4 = likely, and 5 = very likely. People who responded ‘very unlikely’, ‘unlikely’, or ‘neutral’ were defined as having vaccine hesitancy.

#### 2.3.4. Perceptions Related to COVID-19, Second COVID-19 Vaccine Booster Dose and Vaccine Fatigue

Regarding perceptions about COVID-19, we added one new item “How high is your chance of having close contact with people having COVID-19?” to a validated item measuring perceived susceptibility to COVID-19 [36,38], and formed the Perceived Susceptibility Scale. The Cronbach’s alpha of the Perceived Susceptibility Scale was 0.63. The Perceived Severity Scale was constructed for this study by summing up three items measuring perceived consequences of COVID-19 infection (i.e., chance of having severe illness, negatively affecting income and health of other family members) (response categories: 1 = low, 2 = neutral, 3 = high). The Cronbach’s alpha of the Perceived Severity Scale was 0.70. We adapted the validated scales or items to measure perceived benefits, perceived barriers, cues to action, and perceived self-efficacy related to the second COVID-19 vaccine booster dose [39]. The phrase “COVID-19 vaccine booster dose” in the original measurements was replaced by “second COVID-19 vaccine booster dose” in this study [39]. 

One single item was constructed to measure vaccine fatigue (i.e., you are tired of receiving repeated COVID-19 vaccination). The response categories for the above items were 1 = disagree, 2 = neutral, 3 = agree.

#### 2.3.5. Interpersonal Level Variables

Validated items were adapted to measure the frequency of exposure to the following contents on TV, radio, newspaper, and Internet: (1) people infected with COVID-19 after receiving three doses of COVID-19 vaccines, and (2) people recovered from COVID-19 without seeking medical consultation [37]. We used a validated measurement to measure thoughtful consideration about the veracity of COVID-19-specific information [45].

### 2.4. Sample Size Planning

The targeted sample size of this study was 360. We assumed 50% of the elderly population intended to receive a second COVID-19 vaccine booster dose. With the assumption of the prevalence of behavioral intention in the reference group (without a facilitating condition) to be 10–40%, this targeted sample size could be able to detect the smallest odds of 1.76 between individuals with or without a facilitating condition (Power: 0.80, alpha value: 0.05; PASS 11.0, NCSS LLC, Kaysville, UT, USA). The same sample size planning approach was used in published studies [41].

### 2.5. Statistical Analyses

We followed the statistical methods used in a number of published studies [40,41,46]. The frequency distribution of all variables was established. The mean and standard deviation (SD) of the items and scales representing perceptions related to the second COVID-19 booster dose were also calculated. Univariate logistic regression models first assessed the significance of associations between background characteristics and the dependent variable (i.e., vaccine hesitancy). We then fitted a single logistic regression model involving all significant background characteristics and one independent variable of interest at a time. Crude odds ratio (OR), adjusted odds ratios (AOR), and their 95% confidence intervals (CI) were reported. Analyses were performed using SPSS (version 26.0; IBM, Armonk, NY, USA). *p* < 0.05 was considered statistically significant.

## 3. Results

### 3.1. Background Characteristics of the Participants

About half of the participants were 65–69 years (49.2%) and female (60.8%). The majority of them were married or cohabited with a partner (74.6%), did not receive tertiary education (89.2%), were without a full-time or part-time job (86.2%), and had a monthly household income below HKD 20,000 (USD 2580) (73.8%). Over half had at least one chronic condition (60.3%). The most prevalent chronic condition was hypertension (46.8%), followed by diabetes mellitus (18.9%) and chronic cardiovascular diseases (10.8%). Among the participants, 25.4% reported a history of COVID-19 infection. At the survey time, 66.2% had received a seasonal influenza vaccination and 28.6% had received pneumococcal vaccination in their lifetime (Table 1).

### 3.2. Second COVID-19 Vaccine Booster Dose Uptake and Vaccine Hesitancy to Receive the Second COVID-19 Vaccine Booster Dose

Among the participants, 3.5% (n = 13) received a second COVID-19 vaccine booster dose. More participants chose Comirnaty (n = 8, 61.5%) rather than CoronaVac (n = 5, 38.5%) as their second booster dose. The majority reported no side effects (46.2%), and the side effects were very mild or mild (46.2%) with the second booster dose. The prevalence of hesitancy to receive the second booster dose was 52.4% (Table 2).

### 3.3. Perceptions Related to COVID-19 and the Second COVID-19 Vaccine Booster Dose

For perceived susceptibility, 3.8% and 24.0% of the participants perceived the chance of COVID-19 infection and having another wave of COVID-19 outbreak in Hong Kong was high/very high, respectively. For perceived severity, 21.1%, 10.5%, and 33.0% perceived the chance of having severe illness, financial difficulties caused by COVID-19, and transmitting COVID-19 to family members was high/very high, respectively (Table 2).

Regarding perceptions related to the second COVID-19 vaccine booster dose, the majority had positive attitudes toward the second COVID-19 vaccine booster dose, such as the belief that a second booster dose was highly effective in preventing severe consequences of COVID-19 (73.8%), could maintain their antibody level and strengthen the protection against COVID-19 (59.2%), and was highly effective in protecting them from the Omicron variant (47.8%). About 34.1% and 20.0% were concerned that the presence of chronic diseases would decrease the protection of the second booster dose and that the duration of protection offered by the second booster dose was shorter among people with older age, respectively. About a quarter (27.0%) reported that their family doctors would suggest them to receive the second booster dose and less than half (43.8%) reported that their family members would suggest them to receive the second booster dose. The majority (86.8%) were confident to receive the second booster dose. However, over half (54.3%) reported that they were tired of receiving repeated COVID-19 vaccination (vaccine fatigue) (Table 2).

### 3.4. Factors Associated with Hesitancy to Receive the Second COVID-19 Vaccine Booster Dose

Females had a higher level of hesitancy to receive the second COVID-19 vaccine booster dose than males (AOR: 1.92, 95%CI: 1.26, 2.94) (Table 3). After adjustment for gender, those with more perceived barriers to receive the second booster dose (AOR: 1.23, 95%CI: 1.12, 1.34) and who were tired of receiving repeated COVID-19 vaccinati (vaccine fatigue) (AOR: 1.90, 95%CI: 1.52, 2.38) were more likely to have a hesitancy to receive the second booster dose. Perceived benefits of the second booster dose (AOR: 0.50, 95%CI: 0.42, 0.60), cues to action (AOR: 0.39, 95%CI: 0.30, 0.52), and higher self-efficacy (AOR: 0.37, 95%CI: 0.21, 0.66) were associated with lower hesitancy to receive the second booster dose (Table 4).

Key findings of the study:

- Half of the participants (52.4%) were hesitant to receive the second COVID-19 vaccine booster dose.
- Perceived benefits, cues to action, and perceived self-efficacy of receiving the second booster dose were associated with lower vaccine hesitancy.
- Perceived barriers and vaccine fatigue (tired of receiving repeated COVID-19 vaccination) were associated with higher vaccine hesitancy.

## 4. Discussion

To our knowledge, this is one of the first studies to examine the hesitancy to receive the second COVID-19 vaccine booster dose and its associated factors among older adults in China. Factors at the individual level were determinants of hesitancy to receive the second booster dose. The findings provided a knowledge basis to develop tailored behavioral interventions to reduce vaccine hesitancy to receive the second booster dose among older adults.

In this study, the socioeconomic and educational status of the study populations were similar to the general population aged 65 years or above reported by the Hong Kong government [47,48,49]. Half of the older adults (52.4%) were hesitant to receive the second COVID-19 vaccine booster dose. Older adults were the most vulnerable age group during the COVID-19 pandemic [2]. Given the promising effectiveness of the second booster dose in preventing severe consequences and deaths associated with COVID-19, there is a strong need to decrease the vaccine hesitancy to receive the second booster dose among older adults in Hong Kong. This hesitancy rate was much higher than that of the first booster dose found in China (17.2–18.3%) [30,32] and the second booster dose among the general population in Greece (38.1%) [33]. One of the reasons for this high vaccine hesitancy may be due to vaccine fatigue. In our findings, over half (54.3%) of the older adults agreed that they were tired of receiving repeated COVID-19 vaccination. During the pandemic, policies and recommendations related to COVID-19 vaccination have been changing rapidly. Such changes might confuse the general public. In the United States, many people were confused about the meaning of “fully-vaccinated” when booster doses became part of the vaccination regimen [35]. Such confusion further increased when health authorities recommended the second booster dose. It is possible that many older adults in Hong Kong would have similar confusion. The confusion increases vaccine fatigue and results in a higher hesitancy to receive a second COVID-19 vaccine booster dose. In order to address vaccine fatigue, greater investment in vaccine technologies is needed to make more user-friendly vaccines available with better overall efficacy, longer duration of protection, and simpler logistics associated with dose administration. On the other hand, health authorities may convey the message that taking booster doses is to maintain their protection level against COVID-19, which is similar to taking up influenza vaccination every year. 

The findings provided some empirical insights for developing interventions to decrease vaccine hesitancy of receiving the second COVID-19 vaccine booster dose. The majority of participants perceived some benefits of receiving the second COVID-19 booster dose, notably that it was highly effective in preventing severe consequences of COVID-19 (73.8%) and it could maintain their antibody level and strengthen the protection against COVID-19 (59.2%). Those who scored higher in perceived benefit were less hesitant to receive the second booster dose. Future health promotion campaigns should strengthen such beliefs among older adults. About one-third of participants (34.1%) had concerns about the presence of chronic diseases that would decrease the protection of the second booster dose. One-fifth of the participants (20.0%) had concerns about the shorter duration of protection offered by the second booster dose among older adults. It is important to address such concerns among older adults as they were significantly associated with higher hesitancy to receive the second booster dose. Health promotions should clearly convey a message to the older adults that receiving the second booster dose could protect people with chronic diseases and there is no significant difference between the duration of protection for older adults and younger adults. Perceived cue to action and self-efficacy were both facilitators. Future programs should encourage significant others, such as family doctors and members, to give reminders to receive the second booster dose as a strong cue to action. To increase self-efficacy, creating a promotional video including a role model (i.e., older adults) demonstrating the specific procedures to take up the second booster dose and facilitating them to form an action plan are potentially useful strategies. 

This study had several limitations. First, selection bias existed due to non-response. The refusals might have different characteristics compared to the participants and we were not able to know how non-response would affect the results. However, our response rate was comparable to random telephone surveys on vaccination behaviors among community-dwelling older adults of previous studies [40,41,42]. Second, data were self-reported, and verification was not feasible. Social desirability bias and recall bias existed. Third, it was possible that community-dwelling older adults would perceive a stronger need to receive a second booster dose due to the increase in daily-confirmed COVID-19 cases from the start of the study (11 May 2022) to the end of the study (11 July 2022). Fourth, causal relationships could not be established, as our study design was a cross-sectional study. Finally, this study was conducted in one Chinese city and did not include older residents in residential care homes. The participants could not represent all older adults in China. Therefore, the generalization of the results to other parts of China should be made with caution.

## 5. Conclusions

Community-dwelling older adults aged 65 years or above in Hong Kong reported a high level of vaccine hesitancy to receive the second COVID-19 vaccine booster dose. Health authorities should address vaccine fatigue and concerns about the interaction between the presence of chronic diseases and the second booster dose. Strengthening perceived benefits, involving significant others of older adults, and increasing perceived self-efficacy to receive the second booster dose might also be useful strategies in this age group.

## Figures and Tables

**Table 1:** Background characteristics of the participants.

Characteristics	N	%
**Sociodemographic characteristics**		
Age, years		
65–69	182	49.2
70–74	125	33.8
75 or above	63	17.0
Gender		
Male	145	39.2
Female	225	60.8
Relationship status		
Currently single	94	25.4
Married or cohabited with a partner	276	74.6
Education level		
Primary or below	157	42.4
Secondary	173	46.8
Tertiary or above	40	10.8
Current employment status		
Unemployed/retired/housewife	319	86.2
Full-time/part-time	51	13.8
Monthly household income, HK$ (US$)		
<20,000 (2580)	273	73.8
20,000 (2580) or above	49	13.2
Refuse to disclose	48	13.0
Receiving Comprehensive Social Security Assistance (CSSA)		
No	197	53.2
Yes	173	46.8
Living alone		
No	304	82.2
Yes	66	17.8
**Health conditions**		
Presence of chronic conditions, yes		
Hypertension	173	46.8
Chronic cardiovascular diseases	40	10.8
Chronic lung diseases	6	1.6
Chronic liver diseases	8	2.2
Chronic kidney diseases	2	0.5
Diabetes Mellitus	70	18.9
Any of the above	223	60.3
History of COVID-19 infection		
No	276	74.6
Yes	94	25.4
History of seasonal influenza vaccination		
No	125	33.8
Yes	245	66.2
History of pneumococcal vaccination		
No	264	71.4
Yes	106	28.6

**Table 2:** Hesitancy and attitudes toward the second COVID-19 booster dose.

Characteristics	N	%
**COVID-19 vaccination uptake**		
Number of doses of COVID-19 vaccination		
0	16	4.3
1	13	3.5
2	123	33.2
3	205	55.4
4	13	3.5
Types of second COVID-19 booster dose (among 13 participants who had received the second booster dose)		
CoronaVac	5	38.5
Comirnaty	8	61.5
Side-effects of the second COVID-19 vaccine booster dose (among 13 participants who had received the second booster dose)		
Not at all	6	46.2
Very mild	3	23.1
Mild	3	23.1
Moderate	0	0.0
Severe	0	0.0
Very severe	1	7.7
Likelihood to receive the second COVID-19 booster dose in the future 6 months (among 357 participants who had not received the second booster dose)		
Very unlikely/unlikely/neutral	187	52.4
Likely/very likely	170	47.6
**Perceptions related to COVID-19 and the second booster dose**		
Perceived susceptibility to COVID-19, high/very high		
Chance of COVID-19 infection	14	3.8
Chance of having another wave of COVID-19 outbreak in Hong Kong	89	24.0
Perceived Susceptibility Scale ^1^ Mean (SD)	5.3	(1.3)
Median (IQR)	5.0	(4, 6)
Perceived severity of COVID-19, high		
Chance of having severe illness caused by COVID-19	78	21.1
Chance of having financial difficulties caused by COVID-19	39	10.5
Chance of transmitting COVID-19 to family members	122	33.0
Perceived Severity Scale ^2^ Mean (SD)	5.3	(1.7)
Median (IQR)	5.0	(4, 6)
Perceived benefit of the second COVID-19 vaccine booster dose, agree		
Receiving a second booster dose can maintain your antibody level and strengthen the protection against COVID-19	219	59.2
A second booster dose is highly effective in protecting from the Omicron variant	177	47.8
A second booster dose is highly effective in preventing severe consequences of COVID-19	273	73.8
Perceived Benefit Scale ^3^ Mean (SD)	7.5	(1.6)
Median (IQR)	8.0	(6, 9)
Perceived barrier to receive the second COVID-19 vaccine booster dose, agree		
The protection offered by the second booster dose is weaker among people with older age	61	16.5
The level of side effects of the second booster dose is more severe among people with older age	63	17.0
The duration of protection offered by the second booster dose is shorter among people with older age	74	20.0
Presence of chronic diseases would decrease the protection of the second booster dose	126	34.1
Perceived Barrier Scale ^4^ Mean (SD)	7.3	(2.4)
Median (IQR)	8.0	(5, 9)
Cue to action related to the second COVID-19 vaccine booster dose, agree		
Your family doctors would suggest you to receive the second booster dose	100	27.0
Your family members would suggest you to receive the second booster dose	162	43.8
Cue to Action Scale ^5^ Mean (SD)	4.6	(0.9)
Median (IQR)	4.0	(4, 5)
Perceived self-efficacy to take up the second COVID-19 vaccine booster dose, agree		
You are confident to receive the second booster dose	321	86.8
Item score, mean (SD)	2.8	0.5
Median (IQR)	3.0	(3, 3)
Tired of receiving repeated COVID-19 vaccination (vaccine fatigue), agree	201	54.3
Item score, mean (SD)	2.1	(1.0)
Median (IQR)	3.0	(1, 3)
**Frequency of exposure to the following contents on TV, radio, newspaper and Internet in the past month**		
People infected with COVID-19 after receiving three doses of COVID-19 vaccines, sometimes/always	234	63.3
Item score, mean (SD)	2.7	(1.0)
Median (IQR)	3.0	(2, 3)
People recovered from COVID-19 without seeking medical consultation, sometimes/always	179	62.2
Item score, mean (SD)	2.7	(1.0)
Median (IQR)	3.0	(2, 3)
Thoughtful consideration about the veracity of COVID-19-specific information, sometimes/always	198	53.5
Item score, mean (SD)	2.5	(1.1)
Median (IQR)	3.0	(2, 4)

^1^ Perceived Susceptibility Scale, 2 items, Cronbach’s alpha: 0.63. ^2^ Perceived Severity Scale, 3 items, Cronbach’s alpha: 0.70. ^3^ Perceived Benefit Scale. 3 items, Cronbach’s alpha: 0.76. ^4^ Perceived Barrier Scale, 4 items, Cronbach’s alpha: 0.81. ^5^ Cue to Action Scale, 2 items, Cronbach’s alpha: 0.68.

**Table 3:** Associations between background characteristics and hesitancy to receive the second COVID-19 vaccine booster dose.

Characteristics	OR (95%CI)	*p* Values
**Sociodemographic characteristics**		
Age, years		
65–69	1.0	
70–74	0.93 (0.59, 1.47)	0.76
75 or above	1.15 (0.65, 2.04)	0.64
Gender		
Male	1.0	
Female	1.92 (1.26, 2.94)	0.002
Relationship status		
Currently single	1.0	
Married or cohabited with a partner	0.82 (0.51, 1.31)	0.41
Education level		
Primary or below	1.0	
Secondary	0.71 (0.46, 1.09)	0.12
Tertiary or above	0.58 (0.29, 1.17)	0.13
Current employment status		
Unemployed/retired/housewife	1.0	
Full-time/part-time	0.65 (0.35, 1.18)	0.15
Monthly household income, HK$ (US$)		
<20,000 (2580)	1.0	
20,000 (2580) or above	0.55 (0.30, 1.03)	0.06
Refuse to disclose	0.67 (0.36, 1.26)	0.21
Receiving Comprehensive Social Security Assistance (CSSA)		
No	1.0	
Yes	0.61 (0.28, 1.34)	0.22
Living alone		
No	1.0	
Yes	1.64 (0.96, 2.83)	0.07
**Health conditions**		
Presence of any chronic conditions		
No	1.0	
Yes	0.89 (0.58, 1.34)	0.57
History of COVID-19 infection		
No	1.0	
Yes	1.37 (0.86, 2.19)	0.19
History of seasonal influenza vaccination		
No	1.0	
Yes	0.68 (0.44, 1.06)	0.09
History of pneumococcal vaccination		
No	1.0	
Yes	0.79 (0.50, 1.23)	0.29

OR: crude odds ratios. CI: confidence interval.

**Table 4:** Factors associated with hesitancy to receive the second COVID-19 vaccine booster dose.

	OR (95%CI)	*p* Values	AOR (95%CI)	*p* Values
**Perceptions related to COVID-19 and the second booster dose**				
Perceived Susceptibility Scale	0.97 (0.83, 1.14)	0.74	0.94 (0.80, 1.11)	0.49
Perceived Severity Scale	1.00 (0.89, 1.12)	0.95	0.99 (0.88, 1.11)	0.83
Perceived Benefit Scale	0.52 (0.44, 0.61)	<0.001	0.50 (0.42, 0.60)	<0.001
Perceived Barrier Scale	1.21 (1.11, 1.32)	<0.001	1.23 (1.12, 1.34)	<0.001
Cue to Action Scale	0.41 (0.31, 0.53)	<0.001	0.39 (0.30, 0.52)	<0.001
Perceived self-efficacy	0.37 (0.21, 0.64)	<0.001	0.37 (0.21, 0.66)	0.001
Tired of receiving repeated COVID-19 vaccination (vaccine fatigue)	1.97 (1.58, 2.46)	<0.001	1.90 (1.52, 2.38)	<0.001
**Frequency of exposure to the following contents on TV, radio, newspaper and Internet in the past month**				
People infected with COVID-19 after receiving three doses of COVID-19 vaccines	1.05 (0.85, 1.28)	0.67	1.02 (0.83, 1.53)	0.83
People recovered from COVID-19 without seeking medical consultation	0.95 (0.78, 1.17)	0.95	0.92 (0.74, 1.13)	0.41
Thoughtful consideration about the veracity of COVID-19-specific information	0.94 (0.78, 1.13)	0.51	0.91 (0.76, 1.10)	0.32

OR: crude odds ratios. AOR: adjusted odds ratios, odds ratios adjusted for gender. CI: confidence interval.

## Data Availability

The data presented in this study are available from the corresponding author upon request. The data are not publicly available as they contain personal behaviours.
